# Neural inhibition as implemented by an actor-critic model involves the human dorsal striatum and ventral tegmental area

**DOI:** 10.1038/s41598-024-56161-8

**Published:** 2024-03-16

**Authors:** Ana Araújo, Isabel Catarina Duarte, Teresa Sousa, Joana Oliveira, Ana Telma Pereira, António Macedo, Miguel Castelo-Branco

**Affiliations:** 1 Coimbra Institute for Biomedical Imaging and Translational Research (CIBIT), University of Coimbra, Coimbra, Portugal; 2 Institute for Nuclear Sciences Applied to Health (ICNAS), University of Coimbra, Coimbra, Portugal; 3 Institute of Psychological Medicine, Faculty of Medicine, University of Coimbra, Coimbra, Portugal; 4 Faculty of Medicine, University of Coimbra, Coimbra, Portugal; 5 Department of Psychiatry, Coimbra Hospital and University Centre, Coimbra, Portugal

**Keywords:** Neuroscience, Cognitive neuroscience, Neural circuits

## Abstract

Inhibition is implicated across virtually all human experiences. As a trade-off of being very efficient, this executive function is also prone to many errors. Rodent and computational studies show that midbrain regions play crucial roles during errors by sending dopaminergic learning signals to the basal ganglia for behavioural adjustment. However, the parallels between animal and human neural anatomy and function are not determined. We scanned human adults while they performed an fMRI inhibitory task requiring trial-and-error learning. Guided by an actor-critic model, our results implicate the dorsal striatum and the ventral tegmental area as the actor and the critic, respectively. Using a multilevel and dimensional approach, we also demonstrate a link between midbrain and striatum circuit activity, inhibitory performance, and self-reported autistic and obsessive–compulsive subclinical traits.

## Introduction

In the course of their daily lives, individuals often encounter complex and dynamic situations demanding the inhibition of conflicting impulses to select the most appropriate actions. While intact inhibition is associated with better physical and mental health^1^, failures of this process underly a range of neurodevelopmental disorders such as autism spectrum disorder (ASD) and obsessive–compulsive disorder (OCD)^2–4^. Even in healthy individuals, inhibitory processes are very susceptible to failures such as inadvertently running a semaphore red light^5^. In these scenarios, the ability to learn from errors and adjust actions accordingly is essential for adaptive behaviour^6,7^.

Despite its multifaceted nature, inhibition has often been assessed as a unitary construct^8^. Even paradigms that dissociate inhibition into distinct domains lack a consensual explanatory framework^4^. This picture hinders the fine-grained understanding of the potentially distinct inhibition facets and their links with the underlying neurobiology^2^. To mitigate these challenges, we assumed that inhibition requires learning, and used a functional magnetic resonance imaging (fMRI) approach to isolate the neural processes underlying successful and failed inhibition, with a specific focus on the behavioural strategy. Then, we integrated our results in the context of a well-validated reinforcement learning framework: the actor-critic model^9,10^. Reinforcement learning is a type of learning, in which subjects learn, by trial-and-error, from their own outcomes to improve future performance^10^.

In the actor-critic model^9,10^, the critic estimates the value of each state to predict future reward and then trains the actor to select the specific action that maximizes reward and minimizes negative outcomes. Evidence originating from rodent and computational models implicates the ventral tegmental area (VTA) and the substantia nigra (SN) as the critic^11,12^, and the dorsal striatum as the actor^13^. By learning over time from the midbrain dopamine neuromodulation, the basal ganglia help to select and initiate the best actions from numerous less adaptive possible choices^14–19^. However, investigation of these structures in humans has been rare and predominantly using reward-conditioned tasks. Hence, how the circuits between the midbrain and striatum adjust to trial-and-error learning in the human brain remains largely unknown, and such insights would hold value for understanding the mechanisms underlying inhibitory control.

The primary focus of our work was on the neural correlates of trial-and-error inhibitory learning using the stop-signal task (SST) as a suitable paradigm to investigate this process^20–24^. Participants were instructed to respond to go-signals but to withhold their response on a minority of trials if a stop-signal followed the go-signal. The stop-signal serves a dual purpose as it provides an instruction to withhold an ongoing response and also conveys direct information about the performance outcome^20^. The SST is commonly used to study inhibitory control and, as it generates many errors, also involves learning^21,23^. Errors in the SST are distinct from those in reward tasks, as they arise from performance failures. Such failures require error monitoring and strategy adjustment^21,22^. In the SST literature, it is generally accepted that these two error-related processes are encoded during failed inhibition^21^. The task algorithm is adaptive to keep performance around a 50% error level (“clamp” mechanism)^24^, and participants were informed of this feature. They were also informed that both responding fast on go-trials and inhibiting the button press on stop-trials were equally important and they should discover (learn) the optimal balance between rapid response execution and successful inhibition^24^.

We followed the hypothesis that the behavioural strategy in the SST is largely determined by the interplay between successful and failed inhibition, through successive adjustments and learning. Accordingly, we addressed the role of the midbrain and basal ganglia regions by comparing successful and failed inhibition outcomes. In line with the actor-critic model analogies, we predicted midbrain and basal ganglia recruitment in relation to the appearance of the unexpected stop signal (successful inhibition and failed inhibition). We also expected to find associations between patterns of activity in the actor-critic regions and: (a) participants’ performance (especially in the dorsal striatum, i.e. the actor) and (b) neurobehavioral subclinical traits of ASD and OCD.

## Results

### Self-report and performance data

Participants adhered to the task rules as indicated by the low rate (< 5%) of omission go trials. In around half of the stop trials (51.58 ± 0.67%), participants were able to successfully withhold their response (*Successful Stop*), while in the other half, they failed to stop (*Failed Stop*), which reflects the effective operation of the staircase procedure. The mean reaction time on go trials (GoRT; 612.84 ± 36.19 ms) and stop-signal reaction time (SSRT; 243.98 ± 9.20 ms) were within the normal range^25^. The strategy applied by the group revealed mean response delays (mean stop-signal delay [SSD]) of 367.49 ± 40.61 ms. While the SSRT reflects the final output of the stopping process, the SSD is related to the individual stopping strategy^26^. Given our focus on the trial-and-error learning processes underlying inhibition, for subsequent brain-behaviour analysis, we only report results concerning the SSD. However, it is important to note that we were able to replicate those results for the GoRT, a variable that is intrinsically related to the SSD.

As expected, mean scores of ASD (14.22 ± 1.89) and OCD traits (13.79 ± 8.01) were below the cut-off of each scale.

### Region of interest analysis of basal ganglia and midbrain dopaminergic regions

**Figure 1:**
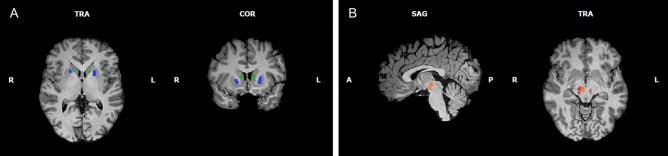
(**A**) Region of interest defined by the intersection between a larger cluster from the whole-brain analysis (*Correct Go* + *Successful Stop* + *Failed Stop* + *Inter-trial Interval* > *Baseline*; RFX, *t*(20) = 5.84, *p-FDR* = 0.001) and anatomical boundaries of the caudate (green) and putamen (blue). (**B**) Region of interest defined by intersections between our whole-brain activations (*Correct Go* + *Successful Stop* + *Failed Stop* + *Inter-trial Interval* > *Baseline*; RFX, *t*(20) = 4.41, *p-FDR* = 0.005) and anatomical boundaries of the substantia nigra (red) and ventral tegmental area (orange).

Our region of interest (ROI) analysis was focused on the basal ganglia and its dopaminergic connections with midbrain regions, based on the hypothesis that implicates these structures in inhibition and reinforcement learning. First, we defined our ROIs based on whole-brain GLM analysis for the contrast *Correct Go* + *Successful Stop* + *Failed Stop* + *Inter-trial Interval* > *Baseline*, which were further validated with anatomical criteria^27,28^. Multiple comparisons correction was set taking into account the localization nature of this independent analysis, such that for smaller structures (those in the midbrain) our threshold was more liberal. This resulted in four ROIs: bilateral caudate’s head, dorsal anterior putamen (Fig. 1A; Table 1), VTA, and SN (Fig. 1B; Table 1). Then, for the resulting ROIs, we extracted the beta values for our two contrasts of interest: successful inhibition (*Successful Stop* > *Correct Go*) and failed inhibition (*Failed Stop* > *Correct Go*). Regions size and peak voxels are described in Table 1. For detailed information regarding the strategy to define our relevant ROIs see the “Methods” section, Supplementary Fig. 1, and Supplementary Table 1.

**Table 1:** List of basal ganglia and midbrain ROIs used for analysis, based on our hypothesis and resulting from the intersection between our whole-brain analysis (*correct go* + *successful stop* + *failed stop* + *inter-trial interval* > *baseline*) and anatomical boundaries.

Region	MNI coordinate (peak)			Nr voxels	*t*	*P*
	*x*	*y*	*z*			
Dorsal Caudate bilateral	16	16	0	1348	8.300	< 1.00 × 10^–6^
Dorsal Putamen bilateral	17	16	− 1	935	7.956	< 1.00 × 10^–6^
Substantia nigra bilateral	−7	− 22	− 11	288	6.146	5.00 × 10^–6^
Lateral VTA bilateral	5	− 16	− 12	604	6.705	2.00 × 10^–6^

### Associations between task strategy and brain activation

To characterize the relationship between participants’ behavioural strategy (SSD) and our ROI activation (beta-values in the dorsal caudate and putamen, SN, and VTA) related to the defined contrasts of interest (successful inhibition [*Successful Stop* > *Correct Go*] and failed inhibition [*Failed Stop* > *Correct Go*]), we applied brain-behaviour correlation analysis. Failed inhibition-related striatal activity correlated with longer SSD in both the caudate (*r* = 0.608, *p*-FDR = 0.018) and putamen (*r* = 0.578; *p*-FDR = 0.036). There was no statistically significant correlation between task performance and successful inhibition activity in either of the analyzed ROIs nor between task performance and SN/VTA failed inhibition activity. For detailed information regarding the correlations between task performance and brain activation see Supplementary Table 2.

### Effect of trial outcome: successful inhibition vs. failed inhibition

**Figure 2:**
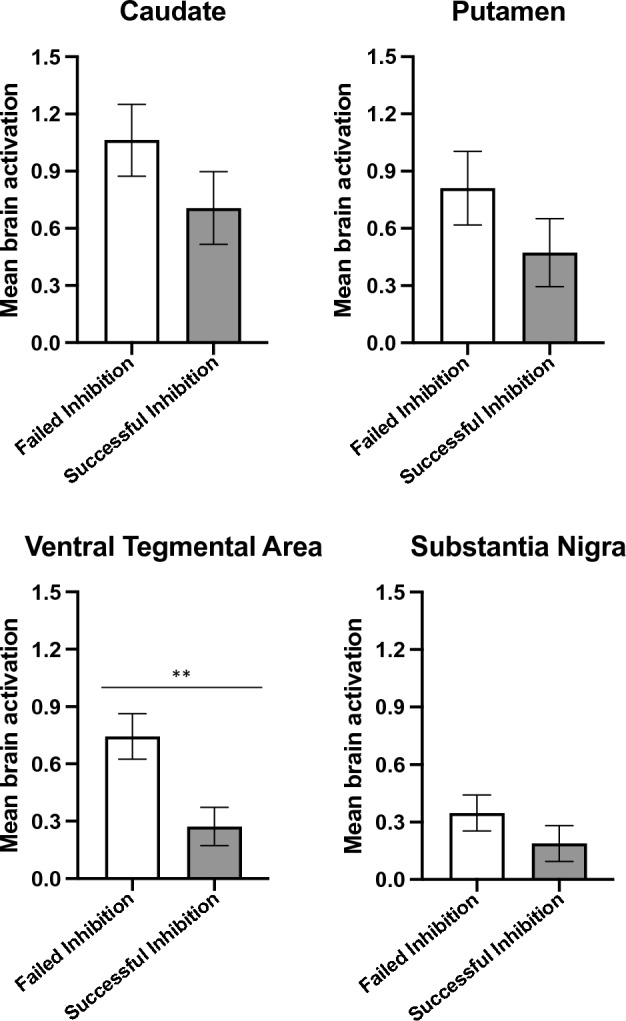
Differences between average ROI responses when comparing successful inhibition and failed inhibition. Error bars represent the standard deviation. There was a significant effect of the ventral tegmental area response on participant performance (*t*(20) = 3.977, *p* < 0.001, Cohen’s d = 0.543, 95% CI [0.356, 1.364]), which therefore was the main region contributing to this effect. **p < 0.01.

To test if failed inhibition (which was presumed to lead to post-error behavioural adjustment) requires higher midbrain-basal ganglia activation than successful inhibition, we used a two-factor (ROI and trial outcome) mixed repeated measures ANOVA. It revealed a significant effect of the trial outcome on brain activation (*Huynh–Feldt F* (6.854, 20) = 17.258, *p* < 0.001). We then performed post hoc paired samples *t*-tests (Supplementary Table 3) to investigate the sources of this effect. As shown in Fig. 2, we found that it resulted mainly from a significantly increased VTA response related to failed inhibition.

Together, these findings point towards the VTA and the striatum as functional regions implicated in error processes that come into action when inhibition fails, with distinct roles.

### Mediation model

**Figure 3:**
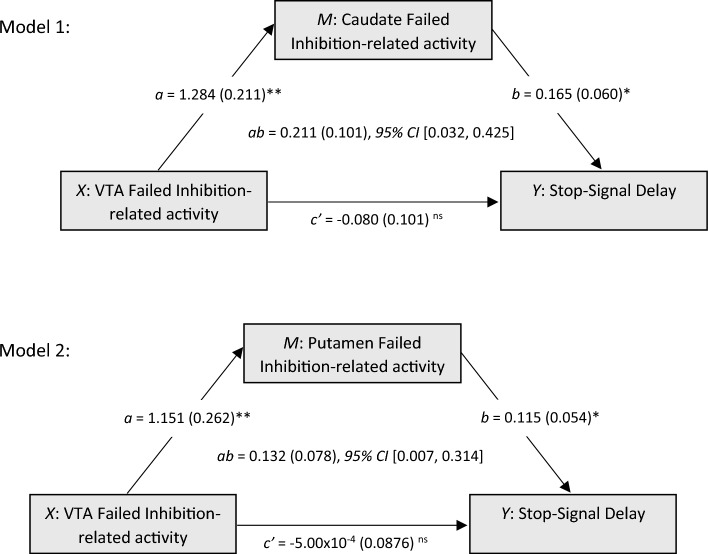
Serial multiple mediation models 1 and 2 show two significant indirect effects of the ventral tegmental area on task strategy (stop-signal delay) mediated by the striatum (caudate or putamen). Numbers represent unstandardized coefficients. Numbers in parentheses represent standard errors. VTA: ventral tegmental area; *X*: predictor variable; *M*: mediator; *Y*: outcome variable; *a*: effect of X on Y; *b*: effect of M on Y; *ab*: indirect effect of X on Y; c’: direct effect of X on Y; CI: confidence interval; **p < 0.01, *p < 0.05, ns: non-significant.

To test midbrain-striatum analogies with the actor-critic model during inhibitory learning, we tested two complementary versions of our hypothesized mediation model (Fig. 3). Specifically, we evaluated the mediation role of the striatum activity (caudate and putamen) on the relationship between the VTA activity and task strategy (SSD values). We found two significant indirect effects related to failed inhibition, namely of: a) the caudate on the relationship between the VTA and the SSD, and b) the putamen on the relationship between the VTA and SSD. The indirect pathway models explained 39.12% (Model 1: *F* = 5.784* p* = 0.012) and 33.45% (Model 2: *F* = 4.523, *p* = 0.026) of the variance of the SSD. We further tested these models with GoRT as outcome variable and we were able to replicate the presented results for the caudate (mediator).

The results of all these models point to a pathway in which the VTA affects task performance through its relationship with the striatum and go against a direct effect of the VTA on behavioural adjustment.

### Functional connectivity analysis

**Figure 4:**
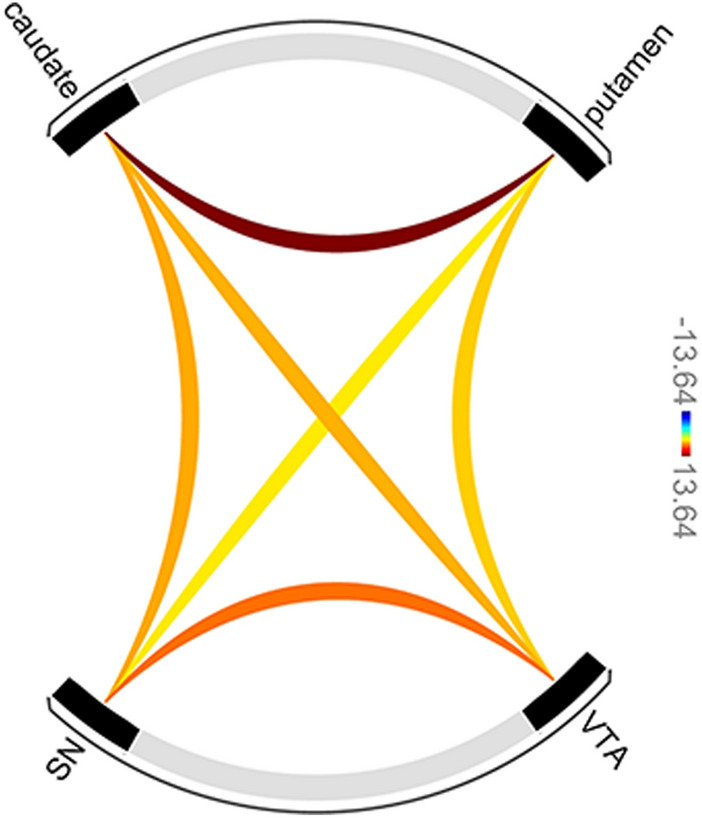
ROI-to-ROI connectivity results, considering data from all subjects, during task performance. The color bar represents the strength of the *t*-statistic when evaluating how much differs connectivity from zero.

In order to investigate the strength of connectivity between our regions of interest during the task, we conducted an ROI-to-ROI correlation analysis, using the ROIs previously defined in the striatum and midbrain^29^. Figure 4 illustrates the significant connections between the nodes of the defined network, represented as a connectome ring. We found that all nodes were positively connected, in particular, within the striatum. Moreover, as shown in Table 2, the caudate was the most significantly connected node. All caudate connections highly differed from zero (as confirmed by the *t*-values), suggesting a central role of this region in receiving and sending information to other key nodes during inhibition.

**Table 2:** Average connectivity values for the selected network during task resolution.

Network connection	*t *(*19*)	*p*-FDR	Effect size
Caudate—Putamen	13.64	1.00 × 10^–7^	0.45
VTA—SN	7.29	2.00 × 10^–6^	0.20
Caudate—SN	5.60	4.20 × 10^–5^	0.10
Caudate—VTA	5.45	4.40 × 10^–5^	0.14
Putamen—VTA	4.64	2.15 × 10^–4^	0.11
Putamen—SN	3.93	9.09 × 10^–4^	0.09

The statistical *t*-values indicate the degree of difference between each connectivity node and zero. *p*-values are corrected for the total number of connections tested.

**Figure 5:**
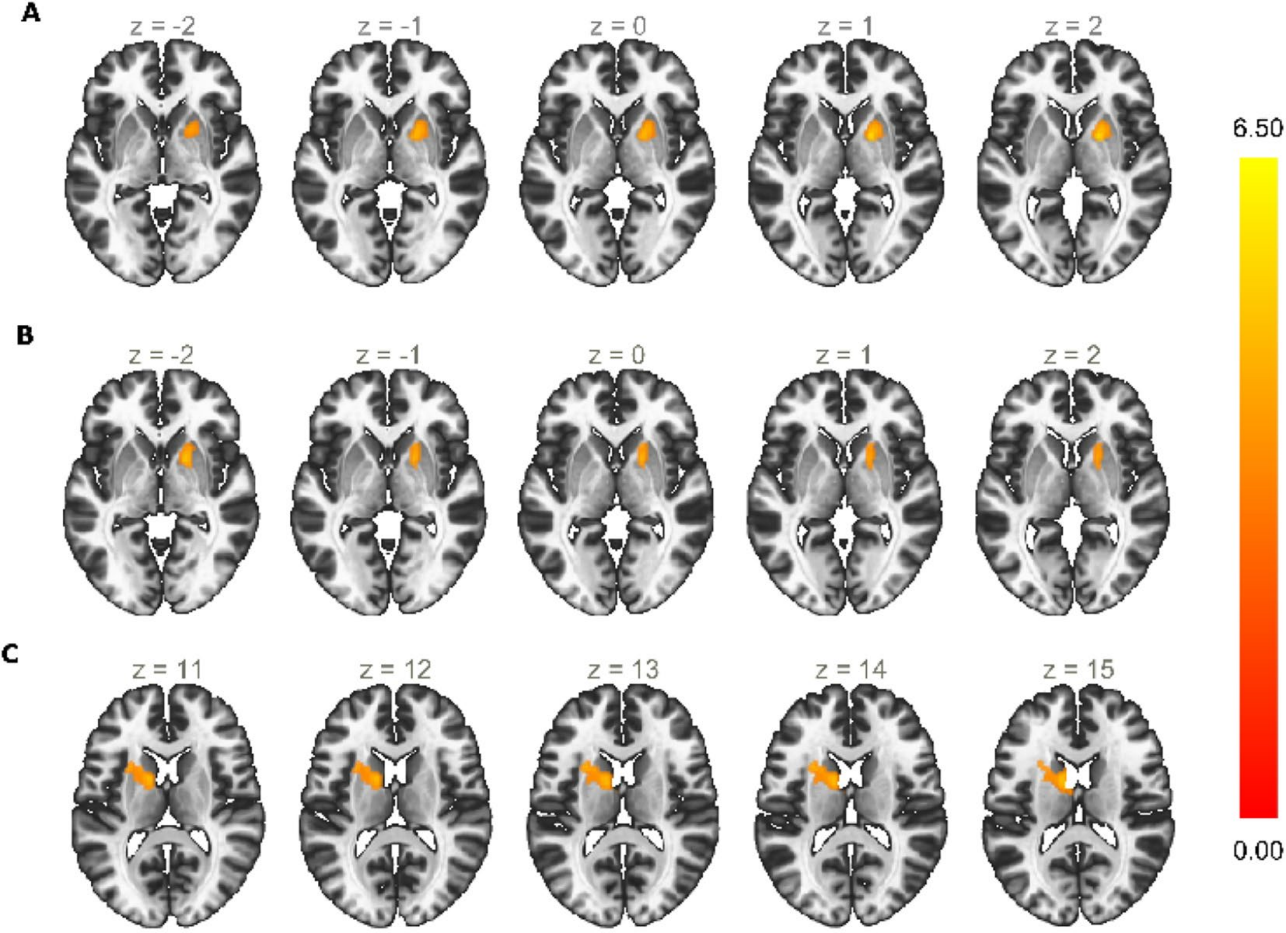
Whole-brain connectivity analysis when considering as seed region the VTA (**A**), caudate (**B**), and putamen (**C**). Color bar represents the strength of the *t*-statistic when evaluating how much differs connectivity between subjects with SSRT lower than the average (higher inhibitory control) and subjects with SSRT higher than the average (lower inhibitory control). The correlation between each seed region and each voxel of the brain is displayed at a voxel threshold of *p* = 0.005 and a cluster threshold of *p-FDR* = 0.05.

We then investigated how was each of these regions connected with all other regions of the brain taking into account the SSRT. To further elucidate the role of the midbrain and the striatum on the inhibitory process, we asked if the connectivity between our seed regions and other voxels in the brain differed between subjects with inhibitory performance higher and lower than the average (Fig. 5). Participants with higher inhibitory performance (SSRT < 243.98 ms) presented higher connectivity between (a) the VTA and a cluster including the right putamen and globus pallidus (*t* = 5.19, *p-FDR* = 6.10 × 10^–4^)), (b) the caudate and a cluster including the right putamen and globus pallidus (*t* = 5.09, *p-FDR* = 7.60 × 10^–4^), and (c) the putamen and a cluster centered in the left caudate (*t* = 5.67, *p-FDR* = 4.50 × 10^–4^).

### Associations between self-report measures and inhibitory-related neural responses

To investigate if the brain regions implicated in inhibitory learning were related to subclinical manifestations of neurodevelopmental spectrum traits, we analyzed correlations between brain activity in our clusters and self-reported measures scores (*n* = 19). Self-reported ASD scores decreased with increasing failed inhibition-related activity in the putamen (*r* = − 0.558, *p* = 0.04) and VTA (*r* = − 0.527, *p* = 0.04, *FDR*-corrected within the symptom dimension), and the same relationship emerged for OCD scores, in the SN (*r* = − 0.570, *p* = 3.5 × 10^–2^), VTA (*r* = − 0.517, *p* = 0.035), and caudate (*r* = − 0.512, *p* = 0.035, *FDR*-corrected within the symptom dimension) related to successful inhibition.

These findings suggest that midbrain and striatal activation during specific stages of the inhibitory process are associated with decreased subclinical neurodevelopmental-spectrum manifestations.

## Discussion

Inhibition is implicated across virtually all our human experiences. As a trade-off of being very efficient, this cognitive function is also prone to many errors. Identifying which neural processes underly the responses elicited by those errors is crucial to understand adaptive and disordered behaviour. In the present study, we invited healthy adults to perform a task that is typically used to probe inhibitory control, and, as it generates many errors, also requires trial-and-error learning and strategy adjustment^21^. We captured two distinct facets of the inhibitory process: successful inhibition and failed inhibition. Considering that next action selection is encoded together with previous outcome monitoring^30^, this approach allowed us to investigate the neural correlates of post-error behavioural adjustment during failed inhibition.

Rodent and computational studies show that the midbrain regions play crucial roles during errors by sending dopaminergic learning signals to the basal ganglia for action selection^11,12,31–38^. However, the human neural processes underlying post-error adjustments remain elusive as there is an obvious lack of investigation in the field^27,39^. Here, we were interested in trial-and-error mechanisms underlying inhibitory learning. This is a crucial function in our daily life as it contributes to withholding irrelevant actions while selecting the most adaptive ones. Guided by an animal and theoretically validated learning model, the actor-critic model^9,10^, we were able to integrate our fMRI results and draw key analogies with human neural functioning. Our main findings were as follows: (a) compared with go trials, both failed and successful inhibition recruited the VTA, SN, and the dorsal striatum (caudate and putamen); (b) increased failed inhibition-related activity in the dorsal striatum was associated with slower responses; (c) failed inhibition, compared with successful inhibition, led to increased activation in our relevant brain structures, in particular, the VTA; (d) during failed inhibition, activity in the dorsal striatum fully mediated the effect of the VTA on the task performance strategy; (e) connectivity analysis further supported the functional relevance of a network including the VTA, SN, caudate and putamen; (f) increased inhibitory activation in both the midbrain and striatum correlated with decreased scores on ASD and OCD subclinical dimensions.

The ability to identify unpredicted stimuli and redirect attention and behavior accordingly is crucial for optimal learning. Our findings show that when compared with the go trials, both successful and failed stop trials recruited regions in the midbrain and dorsal striatum. Specifically, the VTA, SN, caudate’s head, and dorsal anterior putamen activated during the appearance of the unpredicted stop-signals independently of whether the outcome was positive (successful inhibition) or negative (failed inhibition). Reward prediction error theories postulate that dopamine neurons fire to expected or actual rewards^11,12^. However, accumulating animal evidence challenges this view by identifying subpopulations of dopaminergic neurons with heterogeneous response profiles^31,36,37,40–44^. Matsumoto and Hikosaka^35^ recorded monkeys' dopaminergic activity in the VTA and SN during a Pavlovian procedure and found that while ventromedial neurons encoded reward prediction error, neurons located in the dorsolateral region were excited by both reward and punishment, especially when they were unexpected. Our findings indicate that the “warning reaction” evoked by the stop-signal activates the midbrain and dorsal striatum regardless of the specific positive or negative trial outcome. Thus, our results in human males are in line with the existing non-human and experimental literature. In addition, we provide support for the functional specialization of the dorsolateral striatum and midbrain in trial-and-error performance, which is distinct from reward-prediction error processing^11,12^.

It is important to note that, by comparing successful and failed inhibition, we found significant error-related increased activation in the VTA. Based on learning theories, successful inhibition induces a tendency toward repeating the same action, and failed inhibition promotes switching^23,45^. Thus, we may hypothesize that the VTA activation during errors not only reflects the “surprise effect” to the unexpected stop-signal but also encodes an additional operation that induces strategy adjustment. We need to consider the possibility that errors may have resulted from momentary attentional lapses^26^. Although our fMRI design limits conclusions regarding this issue, the generalized task-related default mode network deactivation found in our exploratory whole-brain contrasts indicates that participants were engaged during most of the experiment. Also during failed inhibition, larger activation in the caudate’s head, and dorsal anterior putamen affected the reaction times and was associated with response slowing. Usually, this type of delay on the SST is part of a strategy based on the individual’s motivations to improve the chances of successfully stopping^22–24^. These results are consistent with the role of the basal ganglia in action initiation and performance^19^ and specifically implicate the dorsal striatum in readjusting action-outcome associations during errors^6^. Verharen et al.^44^ found that the stimulation of the rat D2 receptors in the dorsolateral striatum promoted explorative choice behaviour and led to response time increases. Similarly, in our study, participants with higher error-related activity in the dorsal striatum presented longer and more delayed reaction times. It is plausible to presume that striatal activation during errors leads to post-error slowing, reflecting the choice to switch to an explorative mode of action. Together, these findings seem to indicate that while the VTA activates to the occurrence of errors as a control-level mechanism, activity in the dorsal striatum is related to post-error instrumental adjustments^46^.

Guided by the actor-critic framework, our mediation models and connectivity analysis corroborated the functional relevance of a pathway linking the VTA to the dorsal striatum (caudate and putamen) leading to strategy adjustment. Our results suggest that VTA identifies the unexpected stop-signal and estimates an error measure based on the mismatch between the required and the current performance. It might thus use this information to “criticize” the dorsal striatum which then plays the actor by operationalizing the required behavioural adjustments. In conditioned learning tasks, the dorsomedial striatum processes stimulus–response associations and motor and cognitive control, and the ventromedial striatum is involved in reward and punishment learning^6,47^. Here, we found a role of the dorsal striatum in instrumental trial-and-error learning that appears to be relatively independent of the affective input coming from the ventral striatum. Alternatively, we may have to consider that activation in the ventral striatum during performance errors was too brief to be detected by the fMRI^21^. Enhanced temporal precision functional neuroimaging techniques might help clarify this question. The same rationale applies to other regions within the basal ganglia such as the subthalamic nucleus and the globus pallidus, whose roles in modulating the direct and indirect pathways have been described^20,48^.

Another extensively studied dichotomy in the basal ganglia is between the caudate and putamen in relation to goal-directed and habitual actions, respectively. There is an ongoing debate about whether they operate in functionally segregated loops^15^ or if there is a dynamic shift between goal-directed and habitual modes^5,49^. Our connectivity data shows that the caudate and putamen were concomitantly engaged and interacted with each other and the midbrain during inhibitory learning. Further, we found stronger connectivity between these regions in individuals exhibiting better inhibitory abilities. This might suggest that the representation of the instrumental outcome in the SST serves both as an outcome underlying goal-directed learning, and it also reinforces the association between the action and the antecedent stimuli as part of a co-occurring habit-related process^6^. Importantly, the caudate showed the highest connectivity scores with the other regions (putamen, SN, and VTA), ascribing a central role to the goal-directed system during our task. These data go against the view that the corticostriatal loops function independently and suggest that inhibition requires cooperation between the goal-directed and habitual systems. Although our fast event-related design used for activity analysis did not allow for separating brain connectivity into failed versus successful stops, this is an interesting question for future studies.

Symptoms in psychiatry are seen as dimensional constructs and it is relatively well accepted that a continuum exists from normal to pathological behavior^50^. The impairment in inhibitory control and the underlying basal ganglia dysregulation has been implicated in both ASD and OCD^3,51^. However, it is unlikely that the same deficit explains distinct phenotypes^52^. Moreover, the exact direction of the brain activity impairments leading to disordered behaviour is inconsistent across studies^51^. Our observations in healthy individuals suggest a protective function of the midbrain and striatum responses regarding subclinical psychiatric symptoms. Autistic traits increased with decreased activation in the putamen, VTA, and SN during failed inhibition, which corresponds to the period when error awareness processes take place. The link between autism and impaired error monitoring is well-documented^53^. Here, we propose that this relationship also encompasses the subclinical spectrum. Failure to activate the SN, VTA, and caudate during successful inhibition was positively correlated with OCD features. This is consistent with the tendency seen in OCD patients to be less goal-oriented and more habitual, at least to positive outcomes^54^. Our results suggest that although distinct, the neural substrates underlying the overlap between ASD and OCD subclinical features in healthy individuals are closely intertwined. Future work in healthy and clinical samples should thus treat inhibition as a multifaceted phenomenon^52^.

In conclusion, we used a multilevel and dimensional approach and demonstrated a link between midbrain and striatal circuit activity, performance on an inhibition task, and self-reported ASD and OCD traits. During instrumental learning, the roles of the dorsal striatum and the VTA were respectively consistent with the actor and the critic postulated by animal models of reinforcement learning. We thus provide evidence for an actor-critic network in the human brain subserving trial-and-error inhibitory learning which is also implicated in subclinical ASD and OCD manifestations. Because this was a male sample and the age range was relatively limited, generalization of the present findings to the female sex and other neurodevelopmental windows, requires further investigation. Nevertheless, our findings highlight that these questions deserve further investigation and that the pathway between the VTA and the dorsal striatum may be of particular interest when studying instrumental performance in reaction and inhibitory tasks.

## Methods

### Participants

The reported analyses included twenty-one healthy right-handed adult males, recruited locally using social media advertising. We decided to exclude female participants to avoid the confounding effect resulting from sex differences in the actor-critic network activation, as it was previously shown^55^. The age of the participants was 29.56 ± 2.22 years (mean ± standard deviation), allowing us to evaluate a neurodevelopmental period in which inhibitory abilities are already well-developed^55^.

We excluded participants with psychiatric or medical disorders affecting brain development, drug/alcohol dependency, history of head injury, abnormal structural MRI scans, and MRI contraindications.

All participants underwent a comprehensive clinical assessment including an interview with the Mini-International Neuropsychiatric Interview for DSM-IV – Portuguese version 5.0.0 (Guterres T, Levy P, Amorim P (1999), unpublished manuscript)^56^. Evaluation with the Wechsler Adult Intelligence Scale – third edition^57,58^ was applied to all except two of the participants who were unable to return to the research center until the end of the recruitment. Those participants were however included in the analysis because showed no signs of intellectual disability and were able to comprehend the task. Detailed information regarding the sample’s characteristics is provided in Supplementary Table 5. Although the relationship between inhibitory control and intelligence remains unclear^59^, we would not expect to find an influence of IQ on task performance and brain activation. This was proven in our analysis.

This study was conducted under the Declaration of Helsinki. We obtained ethical approval from the local Research Ethics Committee (CHUC-089-20) and all participants signed written informed consent after a detailed explanation of the study procedure.

### MRI procedure

The acquisition session comprised one structural and three functional sequences. The task was performed during the last half-hour of an MRI session with a total duration of 1 h. The SST was presented on an LCD monitor (48.5 × 87.8 cm, 1920 × 1080 pixel resolution, 60 Hz refresh rate) which the participants viewed through a mirror mounted above their eyes. The distances from the eye to the top and to the bottom of the screen were 1750 mm and 1825 mm, respectively. Participants' responses were collected via an MRI-compatible response box. All participants used the dominant hand. When necessary, we ensured correction to normal vision using specific magnetic field-compatible eyeglasses.

### Stop-signal task

**Figure 6:**
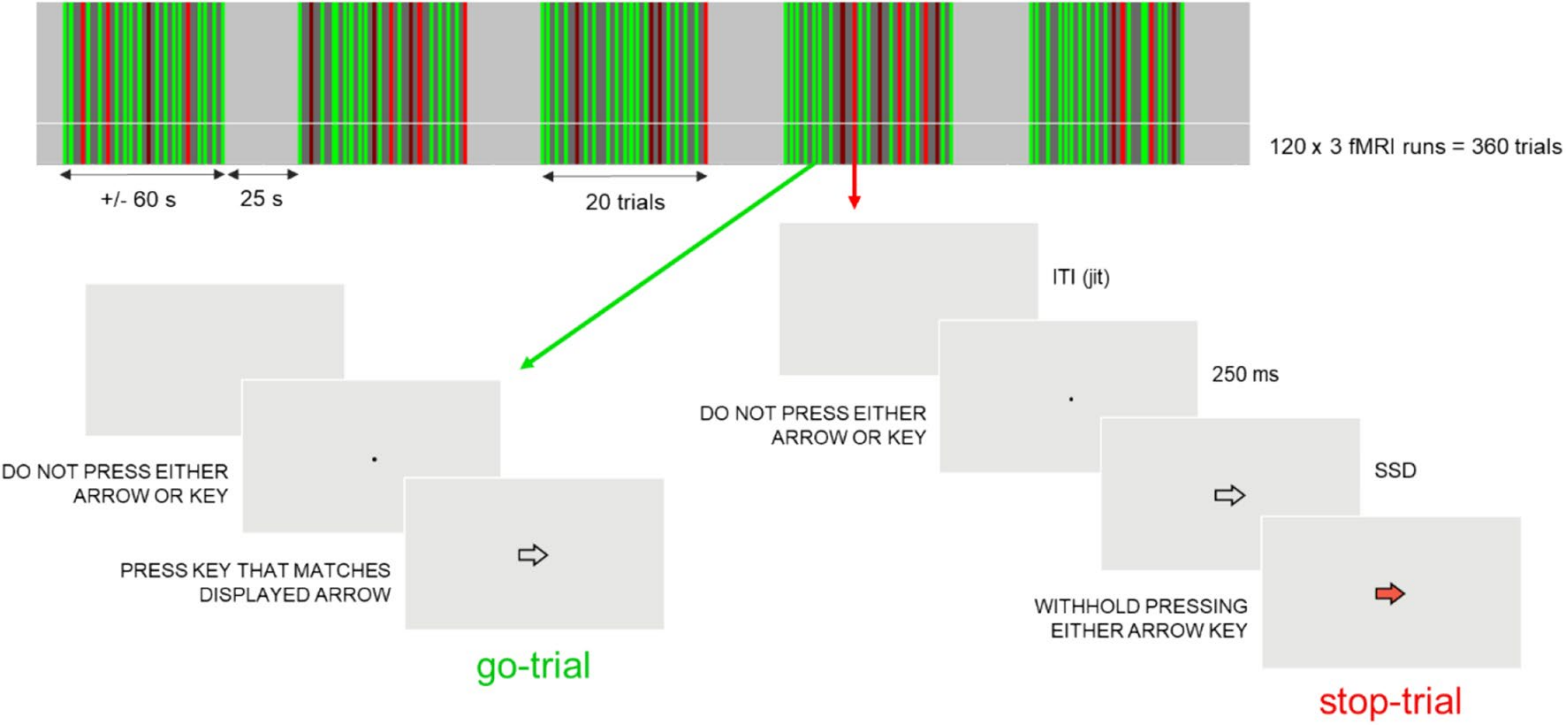
Schematic of the fMRI implementation of the stop-signal task. The task included 3 runs of 120 trials (a total of 360 trials). Within each run, the trials were presented in blocks of 20 trials (60 s) interleaved with a baseline of 25 s. Go trials (75%) began with a fixation dot (250 ms) followed by a go-signal, which was a white arrow either pointing to the right or left side on the screen, instructing participants to press the left or right button of the response. Stop trials (25%) began with a fixation dot (250 ms) followed by the white arrow, which turned red (stop-signal) after a variable period of time (stop-signal delay), instructing the subject to withhold the response. Approximately half of the stop trials could not be stopped, as desired from the implementation of the staircase. The inter-trial interval (the time between the end of the previous trial and the start of the current trial) was jittered between 750 and 2750 ms. *ITI* inter-trial interval, *jit* jittered, *SSD* stop-signal delay.

We developed and presented the SST using Psychophysics Toolbox 3 on MatLab R2019b (The MathWorks, Inc., USA) according to previous consensus guidelines^22–24,60^, as illustrated in Fig. 6. The participants could respond by pressing with the right hand one of two buttons (left or right) of an MRI-compatible response box. We instructed participants to quickly press the right or the left button on go-trials (75% of the total number of trials) and to withhold their response on stop-trials (25% of the total number of trials). We also informed them that both stopping on stop-trials and responding fast on go-trials were equally important; that go-trials were aborted after a fixed period of time; that the task was adaptive to their performance so that at the end of each run the number of *Successful* and *Failed Stop* was approximately equal, and that stopping would not be possible in some of the stop-trials. Participants completed 3 runs of 120 trials (a total of 360 trials). Within each run, blocks of 20 trials were interleaved with baseline periods of 25 s. At the beginning of each trial, a black dot appeared on the screen (250 ms). There were two types of trials. The trial began with an arrow pointing to the right or left side on the screen (go-signal). The arrow was displayed within the central 2.25 degrees of the visual field. In 25% of trials (the stop trials), the arrow in the go-signal turned red (stop-signal). The trials ended at button press or after 1250 ms if the participant did not respond (*Successful Stop* or *Omission Go*). We randomly jittered the mean *Inter-trial Interval* (ITI; the time between the end of the previous trial and the start of the current trial) between 750 and 2750 ms to optimize statistical efficiency. The SSD (the delay between a go-signal and a stop-signal) started at 200 ms and was dynamically changed according to an adaptive staircase procedure, with a 50% performance criterion. If the participant stopped successfully on a stop trial, the SSD latency of the following stop trial increased by 50 ms (up to a maximum of 950 ms), making the task more difficult for the next trial; if the participant failed, the SSD latency decreased by 50 ms (up to a minimum of 100 ms). In this way, at the end of the task, successful stopping was always approximately 50%. Feedback about task performance (RT, number of *Omission go* trials, and % of *Successful Stop*) appeared on the screen at the end of each run. Before entering the MRI scanner, participants performed a training session (240 trials) of the task.

### fMRI acquisition and preprocessing

We acquired functional MRI images using a 3 Tesla Magnetom Prisma Fit scanner (Siemens, Erlangen, Germany), with a 64-channel head coil. The scanning session included one T1-weighted 3D anatomical magnetization prepared rapid acquisition gradient echo pulse sequence, with a repetition time (TR) = 1000 ms, echo time (TE) = 3.5 ms, resolution 2 mm3, flip angle = 7°, 192 slices and field of view (FOV) = 256 × 256 mm. Afterward, we acquired three functional runs using a T2*-weighted gradient echo-planar imaging sequence, with a multi-band acceleration factor of 6, a slice thickness of 2 mm and voxel size 2 mm^2^, 72 interleaved slices without gap, phase encoding direction from anterior to posterior, parallel to the AC-PC line, TR = 1000 ms, TE = 37 ms, flip angle of 68° and FOV of 200 × 200.

Data pre-processing was performed on BrainVoyager 22.0 and 22.4 software (Brain Innovation, Maastricht, Te Netherlands). Pre-processing included slice-scan time correction, 3D head-motion correction, and temporal high-pass filtering (2 cycles per run). To correct for geometrical distortion, we used the COPE plugin^61^. Then, we co-registered the resulting fMRI data and anatomical T1-image, applied a normalization to Montreal Neurological Institute (MNI) standard atlas, and performed spatial smooth using a Gaussian kernel with FWHM of 4 mm. Runs where at some points, the movement exceeded 6 in any axis were excluded from further analysis. Accordingly, we excluded a total of 10 runs. We added motion and physiological signals (respiration and cardiac signals) as confound predictors into the GLM model. PhysIO toolbox for SPM in Matlab^62,63^ was used to create the physiological confound predictors.

### Self-reported questionnaires

All the participants except 1 filled in the Portuguese versions of the following questionnaires. Although none of those are diagnostic per se, higher scores may indicate the need for a more in-depth clinical evaluation^64,65^.

The Autism Spectrum Quotient^64^ is a 50-item self-assessment instrument for measuring the degree to which any adult of normal IQ has traits associated with the autistic spectrum, across five domains ([poor] social skill, [poor] communication, [poor] attention-switching, [exceptional] attention to detail and [poor] imagination). The Autism Spectrum Quotient scores of ≥ 32 indicate the need for further clinical evaluation. The dimensionality of the instrument enables the assessment of ASD traits as a continuous, rather than categorical or diagnostic variables, which was preferable in the present study as we intended to capture the broad autistic phenotype. The Portuguese adaptation of the Autism Spectrum Quotient was performed by a group of experts with experience ASD^66^.

The Obsessive–Compulsive Inventory – Revised^65^ is the short 18-item version of the original 42-item self-assessment questionnaire. The Obsessive–Compulsive Inventory – Revised evaluates the severity of six obsessive–compulsive dimensions (hoarding, checking, ordering, neutralization, cleaning, and obsessions). The instrument was designed for clinical and non-clinical populations. The authors of the original Obsessive–Compulsive Inventory – Revised found an optimal cut-off score of 21 (sensitivity 65.6%; specificity 63.6%). The Portuguese validation presented good psychometric proprieties and confirmed the six related factors structures of the original scale^67^.

### Stop-signal task performance variables

To evaluate performance on the SST^22,23^, we calculated the GoRT, SSD, and the SSRT. The SSRT is a quantitative estimate of the time needed to abort a prepotent (already initiated) response. To estimate the SSRT, we used the integration method from Verbruggen et al.^24^, taking into consideration the independent race model^22^. Shorter SSRTs are indicative of faster reactive stopping^22^, involving a balanced strategy between faster reaction times and the tendency to slow down. While the SSRT reflects the overall efficiency of the stopping process, the SSD determines the individual stopping strategy. Higher SSDs are associated with response slowing, usually applied to increase the likelihood of successful stopping (“speed-accuracy trade-off”).

### Data analysis

For the preprocessing of the neuroimaging data and beta-extraction of the mean activation of our ROIs, we used the Brain Voyager 22.0 and 22.4 software (Brain Innovation, Maastricht, the Netherlands). In the first-level analysis for each subject, we used a general linear model approach. We obtained the predictor’s model by convolution of the time course of each condition with a two-gamma hemodynamic response function. We modeled all regressors with a duration of 1 TR (1 s) from the onset of the stimulus presentation (left and right-pointing arrow). Three types of trial outcomes were distinguished: *Correct Go* (go trials in which participants pressed the correct right or left button), *Successful Stop* (stop trials in which participants inhibited the response of pressing the button), and *Failed Stop* (stop trials in which participants incorrectly pressed the button). Note that *Incorrect Go* (go trials in which participants pressed the wrong-side button) or *Omission Go* trials were not included in the model because were rare events and not all the participants committed those errors.

For the second-level analysis, we applied a random effects (RFX) analysis at the group level to examine the task-related activation (*Correct Go* + *Successful Stop* + *Failed Stop* + *Inter-trial Interval* > *Baseline*) across the whole brain. In line with conceptualizations of inhibition as a dynamic process, this whole-brain exploratory analysis aimed to identify the areas recruited during the SST, encompassing all its required cognitive processes. Then, based on the resulting clusters and guided by current models of inhibition and reinforcement learning, we defined our ROIs in the basal ganglia and dopaminergic midbrain. Supplementary Fig. 1 illustrates the group brain activity map resulting from the contrast between the task and baseline periods. Detailed information regarding the identified clusters is provided in Supplementary Table 1. Contrary to our expectation, neither the subthalamic nucleus nor the nucleus accumbens were in our activation map, even at lower thresholds, so they were excluded from the subsequent region of interest analysis. Both anatomical and functional criteria, taking the intersection of our RFX significant functional activation and anatomical boundaries in the basal ganglia (caudate, putamen, globus pallidus, subthalamic nucleus, SN) and the dopaminergic midbrain (nucleus accumbens, VTA)^28^, were used. To distinguish the clusters within the basal ganglia (RFX, *t*(20) = 5.84, *p-FDR* = 0.001), we applied the MNI-305 Atlas^68^ from the Neuroimaging & Surgical Technologies (McGill University). To identify the SN and midbrain regions, as these are smaller structures difficult to isolate with fMRI^21,68,69^, we used the intersections between our whole-brain maps corrected at a more liberal threshold (RFX, *t*(20) = 4.41, *p-FDR* < 0.005) and two probabilistic atlases of the SN and VTA^27,28^ from the Adcock Lab (Duke University). We confirmed the selected clusters by comparing our peak voxel coordinates with previously outlined anatomical landmarks^27,28^.

Beta-values were extracted from our ROIs for two contrasts of interest: successful inhibition (*Successful Stop* > *Correct Go*) and failed inhibition (*Failed Stop* > *Correct Go*)^70^. In the SST literature, it is generally accepted, that both error monitoring and post-error behavioural adjustment are encoded during failed inhibition^21^. Here, we modeled these two error-related processes as a unitary construct using the contrast of failed inhibition (*Failed Stop* > *Correct Go*). Importantly, while error detection briefly (~ 100 ms) deactivates regions of the salience network, given the low fMRI temporal resolution, this contrast, which joins error monitoring with adjustment, consistently shows positive brain activity within this network^21,71^.

In the subsequent analysis, we used the Statistical Package for Social Sciences, version 26 (SPSS ®, Chicago, IL, USA) to assess the correlations between brain activation, performance variables, and subclinical traits of ASD and OCD. To investigate activation differences between successful and failed inhibition in our ROIs, we performed a two-factor (ROI and trial outcome) mixed repeated measures ANOVA followed by the post hoc paired samples t-Tests. The resulting statistics were corrected to *p-FDR* = 0.05. Then, we conducted a mediation analysis using PROCESS macro (Model 4) for SPSS^72^. The effects were estimated with 5000 bias-corrected bootstrap samples. This approach allowed us to investigate the mediation role of the basal ganglia on the relationship between midbrain activation and task performance. Only the variables that presented significant results in the previous analysis were inserted into the mediation model. Because most of the variables presented normal distributions and homogeneity of variances in Kolmogorov–Smirnov and Levene’s Test, respectively, we used parametric statistics in the analysis.

To further investigate the functional relevance of the circuitry tested we performed a functional connectivity analysis using the CONN toolbox version 22.a^73^ on MATLAB 2020b (MathWorks®). Data were preprocessed using the default minimal preprocessing pipeline. This included functional realignment and unwarp, slice-timing correction, outlier detection, functional normalization into MNI reference space, and spatial smoothing with a 8 mm full width at half maximum Gaussian kernel. Anatomical data preprocessing comprised direct segmentation and normalization, and normalization to MNI space. Subsequently, denoising was performed (by default) to minimize the presence of non-neural noise sources and residual subject motion effects in the BOLD signal. Then, measures of region-to-region functional connectivity (Fisher-transformed Pearson’s correlations) during task performance were computed including all participants’ data. Only the ROIs previously defined (VTA, SN, caudate, and putamen) were considered^29^. Functional connectivity between these brain regions was indexed by a matrix of correlation coefficients reflecting the association between average temporal BOLD time series signals across all voxels in each brain region. Statistical values from these analyses were corrected to *p-FDR* = 0.05. Additionally, we ran a seed‐based connectivity analysis to obtain brain maps of the estimated functional connectivity between each of our ROI and all the voxels in the brain. In first‐level analyses, for each participant, a seed‐based connectivity map was obtained by computing Fisher‐transformed correlation coefficients between the seed ROI and all other voxels. Then, subject‐level results were contrasted between subjects with SSRT lower than the average (SSRT < 243.98 ms, higher inhibitory control) and subjects with SSRT higher than the average (SSRT > 243.98 ms, lower inhibitory control) to investigate whether the connectivity between our seed regions and other brain regions differed between groups. Connectivity values were estimated using a voxel threshold of *p* = 0.005 and a cluster threshold of *p* = 0.05 (*FDR*-corrected). The final functional connectivity group difference maps were converted to *t‐*statistics and overlaid onto the standard MNI template for visualization purposes.

Age and IQ were not included as co-variates in the analysis because no significant correlations were found between these and the other variables in the study (task performance and brain activity). These parameters also did not differ between groups with faster and slower inhibitory performance.

### Supplementary Information

Supplementary Information.

## Data Availability

The datasets generated and/or analysed during the current study are not publicly available due to ethical restrictions but are available from the corresponding author on reasonable request.
