# Supplementary material for: Neural inhibition as implemented by an actor-critic model involves the human dorsal striatum and ventral tegmental area
Source: Sci Rep. 2024 Mar 16;14:6363. doi: 10.1038/s41598-024-56161-8 (PMC10944470; doi:10.1038/s41598-024-56161-8)
Supplement: Supplementary file 1 — Supplementary Information. [file 41598_2024_56161_MOESM1_ESM.docx]

**SUPPLEMENTARY MATERIAL**


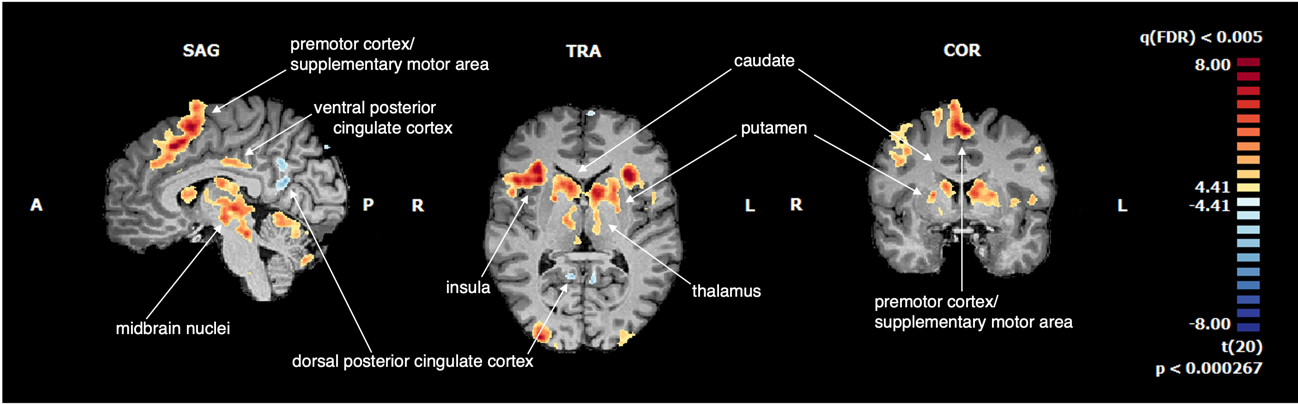


x = 6 y = 4 z = 6

**Supplementary Fig. 1.** Task-related whole-brain activations (*Correct Go + Successful Stop + Failed Stop + Inter-trial Interval > Baseline*; RFX, *t*(20) = 4.41, *p-FDR* = 0.005). Regions in yellow-orange tones represent positive changes in brain activity, and regions in blue tones represent negative changes in brain activity in relation to the baseline. The presented brain maps show activity changes related to the task performance in regions implicated in action planning and inhibition (striatum, thalamus, premotor cortex, supplementary motor area, and frontal eye fields), reward-learning (midbrain nuclei, namely a large cluster including parts of the VTA and SN), salience network (insula), and regions of the executive network (lateral prefrontal cortex). There is also deactivation in the default mode network structures (posterior cingulate cortex, and angular gyrus), which is consistent with the need to direct attention to external stimuli during the task.

**Supplementary Table 1.** List of significant clusters resulting from the whole-brain RFX analysis used the help define the relevant ROI for our main analysis (*t*(20) = 4.41, *p-FDR* = 0.005. Minimum cluster threshold = 50 voxels) when contrasting the task with baseline (*Correct Go* + *Successful Stop* + *Failed Stop* *+ Inter-trial Interval* > *Baseline*).

|  | MNI coordinates (peak) | | |  |  |  | |
| --- | --- | --- | --- | --- | --- | --- | --- |
| Region | *x* | *y* | *z* | Number of voxels | *t* | | *p* |
| Supramarginal gyrus R | 68 | 37 | 29 | 2191 | 6.820 | | 1.00x10^-6^ |
| Motor córtex/ Insula/ Basal ganglia/ Midbrain | 37 | 28 | 0 | 41921 | 9.740 | | < 1.00x10^-6^ |
| Visual córtex/cerebellum R | 43 | -67 | -11 | 8279 | 8.790 | | < 1.00x10^-6^ |
| PFC anterolateral R | 31 | 51 | 29 | 3643 | 6.702 | | 2.00x10^-6^ |
| Secondary visual cortex R | 32 | -95 | 6 | 3356 | 7.740 | | < 1.00x10^-6^ |
| Visuomotor cortex R | 34 | -51 | 47 | 1045 | 6.313 | | 4.00x10^-6^ |
| PFC anterior R | 24 | 56 | -11 | 603 | 6.111 | | 6.00x10^-6^ |
| Premotor/SMA medial bilateral | 5 | 20 | 41 | 11866 | 8.873 | | < 1.00x10^-6^ |
| Cerebellum medial bilateral | 0 | -72 | -14 | 4502 | 6.725 | | 2.00x10^-6^ |
| Dorsal PCC bilateral | -5 | -63 | 28 | 3936 | -8.457 | | < 1.00x10^-6^ |
| Ventral PCC bilateral | 5 | -23 | 30 | 981 | 5.991 | | 7.00x10^-6^ |
| Midbrain inferior | -3 | -42 | -36 | 559 | 6.152 | | 5.00x10^-6^ |
| Visual córtex/ cerebellum L | -35 | -56 | -33 | 12655 | 8.481 | | < 1.00x10^-6^ |
| Visuomotor cortex L | -27 | -70 | 56 | 1287 | 5.413 | | 2.70x10^-5^ |
| Premotor cortex/ SMA L 1 | -28 | -5 | 56 | 418 | 5.724 | | 1.30x10^-5^ |
| PFC anterior L | -35 | 49 | 21 | 757 | 6.619 | | 2.00x10^-6^ |
| Premotor cortex/ SMA L 2 | -41 | -6 | 56 | 1116 | 5.731 | | 1.30x10^-5^ |
| Angular gyrus posterior L | -42 | -75 | 30 | 1821 | -7.076 | | 1.00x10^-6^ |
| Angular gyrus anterior L | -48 | -51 | 34 | 473 | 5.879 | | 9.00x10^-6^ |

**Supplementary Table 2.** Pearson’s correlations between task performance and brain activation related to the task (*n* = 21).

|  | SSD | | GoRT | | SSRT | |
| --- | --- | --- | --- | --- | --- | --- |
| Region | Successful Inhibition | Failed Inhibition | Successful Inhibition | Failed Inhibition | Successful Inhibition | Failed Inhibition |
| Caudate | *r* = 0.316  *p* = 0.196 | *r* = 0.608  *p* = 0.018 | *r* = 0.316  *p* = 0.196 | *r* = 0.555  *p* = 0.027 | *r* = 0.048  *p* = 0.835 | *r* = -0.379  *p* = 0.180 |
| Putamen | *r* = 0.253  *p* = 0.322 | *r* = 0.578  *p* = 0.036 | *r* = 0.257  *p* = 0.322 | *r* = 0.533  *p* = 0.039 | *r* = -0.008  *p* = 0.973 | *r* = -0.281  *p* = 0.322 |
| Ventral tegmental area | *r* = 0.375  *p* = 0.128 | *r* = 0.410  *p* = 0.128 | *r* = 0.397  *p* = 0.128 | *r* = 0.216  *p* = 0.155 | *r* = 0.117  *p* = 0.614 | *r* = -0.232  *p* = 0.374 |
| Substantia nigra | *r* = 0.297  *p* = 0.324 | *r* = 0.322  *p* = 0.324 | *r* = 0.307  *p* = 0.324 | *r* = 0.252  *p* = 0.324 | *r* = 0.017  *p* = 0.943 | *r* = 0.282  *p* = 0.324 |

*p* = 0.05, *FDR*-corrected within each brain region. Note that brain-behaviour correlations are replicated across the performance variables GoRT and SSD. SSD: stop-signal delay; GoRT: mean reaction time on go trials; SSRT: stop signal reaction time.

**Supplementary Table 3.** *Post hoc* comparison of brain activation between trial outcomes (Successful Inhibition vs. Failed Inhibition).

|  | Failed Inhibition | Successful Inhibition | Paired samples t-Test | *Cohen’s d* | 95% CI | |
| --- | --- | --- | --- | --- | --- | --- |
|  |  |  |  |  | Lower | Upper |
| Caudate | *M* = 1.063  *SD* = 0.188 | *M* = 0.706  *SD*= 0.191 | *t* (20) = 1.548  *p* = 0.137 | 1.056 | - 0.106 | 0.774 |
| Putamen | *M* = 0.811  *SD* = 0.193 | *M* = 0.473  *SD* = 0.178 | *t* (20) = 1.445  *p* = 0.164 | 1.073 | - 0.127 | 0.750 |
| VTA | *M* = 0.744  *SD* = 0.119 | *M* = 0.272  *SD* = 0.100 | *t* (20) = 3.977  *p* < 0.001 | 0.543 | 0.356 | 1.364 |
| SN | *M* = 0.347  *SD* = 0.094 | *M* = 0.189  *SD* = 0.094 | *t* (20) = 1.607  *p* = 0.124 | 0.452 | - 0.095 | 0.788 |

M: mean; SD: standard deviation.

**Supplementary Table 4.** Pearson’s correlations between brain activation and self-report measures (*n* = 19).

|  | AQ | | OCI-R | |
| --- | --- | --- | --- | --- |
|  | Successful Inhibition | Failed Inhibition | Successful Inhibition | Failed Inhibition |
| Caudate | *r* = -0.378  *p* = 0.106 | *r* = -0.359  *p* = 0.106 | *r* = -0.512  *p* = 0.035 | *r* = -0.142  *p* = 0.281 |
| Putamen | *r* = -0.312  *p* = 0.129 | r = -0.558  p = 0.040 | *r* = -0.454  *p* = 0.05 | *r* = -0.201  *p* = 0.234 |
| Ventral tegmental area | *r* = -0.199  *p* = 0.207 | *r* = -0.527  *p* = 0.040 | *r* = -0.517  *p* = 0.035 | *r* = -0.425  *p* = 0.052 |
| Substantia nigra | *r* = -0.201  *p* = 0.207 | *r* = -0.405  *p* = 0.106 | *r* = -0.570  *p* = 0.035 | *r* = -0.415  *p* = 0.052 |

*p* = 0.05, *FDR*-corrected within the symptom dimension. AQ: Autism Spectrum Quotient; OCI-R: Obsessive-Compulsive Inventory – Revised.

**Supplementary Table 5.** Sample characteristics (*n* = 21).

|  | *M* ± *SD* |
| --- | --- |
| Age, years | 29.56 ± 2.22 |
| Education, years | 15.19 ± 0.54 |
| IQ-Full Scale | 126.56 ± 2.76 |
| IQ-Verbal | 127.61 ± 2.66 |
| IQ-Performance | 119.28 ± 2.6 |

*M*: mean; *SD*: standard deviation; IQ: Intelligence Quotient
